# Activity Limitation, Health-Care Use, and Unmet Need Among Korean Adults: Cumulative and Conditional Cardiometabolic Care Cascades

**DOI:** 10.3390/healthcare14193198

**Published:** 2026-09-28

**Authors:** Joungmin Kim

**Affiliations:** Healthcare Service School, Soonchunhyang University, 22 Soonchunhyang-ro, Asan-si 31538, Republic of Korea; nicki123@sch.ac.kr

**Keywords:** activity limitation, health-care utilization, unmet health-care need, care cascade, health equity, cardiometabolic disease, Korea National Health and Nutrition Examination Survey

## Abstract

**Highlights:**

**What are the main findings?**

- Korean adults with activity limitation had substantially higher outpatient and inpatient utilization and more than double the unmet health-care need (19.0% vs. 7.7%), yet no higher participation in general health or cancer screening.
- Their greater awareness of hypertension, diabetes, and hypercholesterolemia did not carry through to treatment or control once disease detection was held constant in a conditional cascade.
- Later-stage advantages apparent under cumulative denominators were attenuated under conditional denominators, with no statistically detectable treatment or control differences.

**What are the implications of the main findings?**

- Equity monitoring for adults with activity limitation should extend beyond health-care contact and cumulative coverage to preventive-service uptake and perceived unmet need.
- Reporting both cumulative and conditional cascades can help identify where group differences in chronic-disease management arise.

**Abstract:**

Background: Adults with activity limitation carry a heavier cardiometabolic burden, but whether differences arise in disease detection or subsequent management remains unclear. Cascade studies rarely measure health-care use directly and compute each stage against all prevalent cases—a cumulative formulation that cannot separate detection from later transitions. I assessed utilization, screening, unmet need, and conditional cascade attainment together. Methods: I analyzed the ninth Korea National Health and Nutrition Examination Survey cycle (2022–2024), a nationally representative cross-sectional survey (16,672 adults aged ≥19 years; cardiometabolic and cascade analyses were restricted to adults aged ≥30, and cancer screening to adults aged ≥40). I estimated prevalence ratios (PRs) using survey-weighted Poisson regression, adjusted for age, sex, education, income, smoking, and drinking, with Benjamini–Hochberg false-discovery control across 17 comparisons. I defined cascade stages conditionally and, secondarily, cumulatively. Results: Activity limitation (11.0%) was associated with higher prevalence of hypertension (PR 1.10, 95% confidence interval [CI]1.04–1.17) and diabetes (PR 1.23, 95% CI 1.11–1.37). Outpatient (1.75, 1.63–1.88) and inpatient (2.20, 1.95–2.48) utilization were higher, whereas general health (0.99, 0.95–1.02) and cancer (0.99, 0.95–1.03) screening were not. Unmet need was 19.0% versus 7.7% (2.39, 2.10–2.71). Awareness was higher (1.06–1.14), but no statistically detectable differences were observed in conditional treatment (0.98–1.01) or control (0.98–1.17). Cumulative point estimates for treatment were higher (1.06–1.11), retaining the awareness difference. Conclusions: Higher utilization did not correspond to greater screening or lower unmet need, and higher awareness was not accompanied by statistically detectable advantages in treatment or control. Cumulative and conditional cascades answer different questions; reporting both locates where a management difference arises.

## 1. Introduction

Persons with disabilities and functional limitations experience systematic disadvantage in access to health services and a heavier burden of chronic disease than adults without such limitations [1,2]. The standard interpretation attributes the excess burden to access barriers: physical, communication, and financial obstacles reduce contact with preventive and chronic-disease services, and the resulting shortfall in detection and treatment accumulates as excess disease and poorer control [1].

Two features of the evidence base complicate that interpretation. First, disease occurrence and disease management are distinct outcomes. The care-cascade framework separates them by conditioning on disease being present and decomposing management into sequential stages [3]. However, cascade stages can be defined in two ways that address different questions. A cumulative cascade computes each stage against all prevalent cases and describes the proportion of the diseased population reaching each stage; a conditional cascade computes each stage against attainment of the preceding stage and describes transitions between stages [4]. Because undetected cases are untreated by definition, a group advantage in detection propagates arithmetically through a cumulative cascade; the two formulations are therefore complementary—the cumulative form summarizes how far the diseased population reaches each stage, whereas the conditional form isolates whether treatment differs once disease is known. A recent United States analysis reported higher hypertension prevalence, awareness, and control among adults with disabilities using cumulative denominators [5], extending earlier work confined to prevalence [6]; however, it has not examined whether later-stage advantages persist once detection is held constant.

Second, researchers rarely measure the health-care contact underlying these patterns directly. Analyses commonly infer greater or lesser health-care contact from patterns of cascade indicators, even though utilization, screening participation, and unmet need are measurable quantities. Where measured, adults with functional limitation show distinctive utilization patterns [7], and Korean studies using earlier survey cycles have reported both higher utilization and higher unmet need in this group [8,9]. Measuring these quantities alongside cascade indicators allows patterns of health-care contact and unmet need to be examined directly rather than inferred from cascade attainment alone. This approach allows direct testing of whether high utilization coexists with high unmet need. Two of these quantities are, in part, subjective appraisals rather than clinical events: unmet need is the respondent’s own perception that required care was not obtained, and awareness reflects whether a diagnosis has entered the person’s understanding of their own health. How such appraisals align with objectively measured disease and reported service contact reflects the experience of living with and managing chronic illness, not access alone.

These strands converge on one question—whether the greater health-care contact of adults with activity limitation translates into correspondingly better preventive and cardiometabolic care, or whether it coexists with unchanged screening, higher unmet need, and no advantage in disease management once detection is held constant. To address it, this study used the ninth cycle of the Korea National Health and Nutrition Examination Survey to estimate, in the same respondents, the association of activity limitation with cardiometabolic disease burden, outpatient and inpatient utilization, preventive screening participation, unmet health-care need, and cascade attainment under both cumulative and conditional definitions. South Korea offers an informative setting for this question because near-universal statutory health insurance and organized national screening programs make contact-based financial barriers comparatively small, so any residual disadvantage is unlikely to be explained by coverage alone. I expected that activity limitation would be associated with higher utilization and higher unmet need but not with greater preventive uptake, and that later-stage cascade differences apparent under cumulative denominators would attenuate under conditional denominators. The multivariable models were intended to estimate these associations net of demographic and behavioral confounding rather than to build a predictive model; activity limitation—a self-reported restriction in daily or social activity due to a health problem or a physical or mental disability, distinct from administratively registered disability—is the primary exposure throughout.

## 2. Materials and Methods

This study is reported in accordance with the Strengthening the Reporting of Observational Studies in Epidemiology (STROBE) statement for observational studies [10]; the completed checklist is provided as Supplementary Materials, File S1.

### 2.1. Study Design and Population

The Korea National Health and Nutrition Examination Survey (KNHANES) is an annual, nationally representative cross-sectional survey of the non-institutionalized Korean population conducted by the Korea Disease Control and Prevention Agency under the National Health Promotion Act. It uses a stratified multistage clustered probability design and comprises a health interview, a health examination, and a nutrition survey [11].

Data from the three years of the ninth cycle (2022–2024) were pooled. I excluded years from the eighth cycle because item non-response on the exposure was substantially higher in 2021 than in the subsequent survey years. Of 17,262 respondents aged 19 years or older, all of whom had a valid integrated interview-and-examination weight, 16,672 had non-missing activity-limitation status and formed the analytic sample; 14,757 of these were aged 30 or older and formed the sample for the cardiometabolic prevalence and cascade analyses; the age-30 threshold follows the KDCA national indicator definitions for hypertension, diabetes, and hypercholesterolemia, whereas utilization, screening, and unmet-need analyses used all adults aged 19 or older. The 590 respondents excluded for missing exposure were older than those retained (unweighted mean age 66.4 versus 53.4 years) and did not differ by sex. The Supplementary Materials (Table S1) present the sample flow for each analysis, showing losses to age restriction, outcome missingness, and covariate missingness separately.

### 2.2. Exposure

Activity limitation was ascertained from a survey item (LQ4_00) that asked whether respondents were currently limited in daily life or social activities because of a health problem or a physical or mental disability, coded as present versus absent, with “don’t know” treated as missing. The item is conceptually—though not psychometrically—comparable to the Global Activity Limitation Indicator (GALI) used in European health surveys; the validity evidence synthesized for the GALI [12] does not transfer directly to this single item, so the comparison is conceptual rather than based on established measurement properties. The item differs from disability identifiers built from multi-domain functioning question sets [13]. It is not a measure of registered disability and aggregates heterogeneous underlying causes—physical, sensory, mental, and chronic-disease-related—that cannot be separated here; it does not distinguish severity, duration, type, or cause. The term activity limitation is therefore used throughout in preference to disability, and the findings describe adults reporting activity limitation rather than any administratively defined disability population.

### 2.3. Cardiometabolic Conditions and Cascade Stages

Conditions followed national standard definitions [14]. Hypertension was a mean systolic pressure of 140 mmHg or above, a mean diastolic pressure of 90 mmHg or above, or antihypertensive medication use. Diabetes was defined by the survey’s derived indicator combining fasting glucose of 126 mg/dL or above, glycated hemoglobin of 6.5% or above, and diagnosed disease under treatment. I defined hypercholesterolemia as total cholesterol of 240 mg/dL or above or use of lipid-lowering medication.

Awareness was a prior physician diagnosis. Treatment was antihypertensive medication taken on 20 or more days per month, insulin or an oral hypoglycemic agent, or a lipid-lowering agent. Control was a mean systolic pressure below 140 mmHg and diastolic below 90 mmHg for hypertension, glycated hemoglobin below 6.5% for diabetes, and total cholesterol below 200 mg/dL for hypercholesterolemia, the last two matching the national reporting indicators [14]. Because control thresholds materially affect attainment, I repeated the control analyses using glycated hemoglobin below 7.0% and a commonly used low-density lipoprotein (LDL) cholesterol threshold of 100 mg/dL, taken from the survey’s directly measured LDL variable; I could not apply individualized LDL targets based on cardiovascular risk category.

In the primary analysis, each stage was conditioned on attainment of the preceding stage. For comparison, the cumulative formulation was also reported, in which every stage was computed against all prevalent cases; it should be noted that the national treatment indicator uses the cumulative denominator whereas the national control indicator is computed among treated respondents [14], so the primary specification adopted here departs from the national treatment indicator to separate detection from therapy.

Because prevalence definitions incorporate medication use, and a respondent taking medication is by construction both prevalent and treated, I repeated the awareness analysis using prevalence defined by biomarkers alone. This alternative denominator addresses the incorporation of medication into the prevalence definition, but it selects for currently biomarker-positive cases and therefore excludes treated respondents whose values have normalized; it is not directly comparable with the standard denominator and is reported only as a sensitivity analysis.

### 2.4. Utilization, Preventive Screening, and Unmet Need

Health-care utilization comprised any outpatient visit in the preceding two weeks and any inpatient admission in the preceding year—binary indicators of recent health-care contact that record whether any visit or admission occurred, not the number, intensity, or purpose of visits. The two-week outpatient and one-year inpatient reference periods are the standard KNHANES recall windows, chosen to limit recall error for frequent outpatient events while still capturing rarer inpatient events. These items are therefore described as recent health-care contact rather than as curative or treatment-oriented care. Preventive service uptake comprised receipt of a general health screening examination and receipt of any cancer screening in the preceding two years. Because cancer-specific eligibility varies by age and sex and the survey item aggregates across cancers, the cancer-screening analysis was restricted to adults aged 40 years or older as a pragmatic approximation to the population eligible for the major national screening programs rather than as a full eligibility-based denominator; because the aggregated item combines programs with differing age- and sex-specific eligibility (including cancers screened below age 40), the resulting estimates should be read with corresponding caution.

Unmet health-care need was the survey item recording a perceived need for medical care that went unmet in the preceding year. The item distinguishes respondents who needed care but did not receive it (8.5% of adults, weighted), those who needed care and received it (87.0%), those who reported needing no care (1.8%), and those who did not answer (2.7%). The agency’s own derived indicator assigns the third and fourth categories to missing, so the national unmet-need rate is computed among respondents reporting a care need; that denominator was adopted for the primary analysis, and the whole-population version, in which respondents reporting no need are coded as having no unmet need, is reported as a secondary estimate. The Supplementary Materials (Table S2) give the full frequency table with unweighted counts, weighted percentages, and the profile of each response category.

### 2.5. Covariates and Coding of Survey Skip Patterns

The primary model adjusted for age group, sex, education (four categories), household income quartile, current smoking, and drinking frequency. A stepwise sequence of models is reported: age and sex; plus education and income; plus smoking and drinking (the primary specification); plus health-insurance type, private insurance, and employment status; plus self-rated health. The primary set (age, sex, education, income, smoking, drinking) comprises demographic and behavioral factors that plausibly confound the associations of interest. Health-insurance type, private insurance, and employment were reserved for the fourth model because they may function as mediators or downstream consequences of activity limitation rather than confounders, so adjusting for them risks over-adjustment; self-rated health was reserved for the fifth model for the same reason, as it is plausibly a consequence of activity limitation. Education and income were retained in the primary model because they are established structural determinants of health-care access and conventional confounders of the association between activity limitation and the outcomes examined. Because long-term activity limitation can itself constrain educational attainment and income, however, these variables may lie partly on the causal pathway, so their inclusion may attenuate associations that operate through socioeconomic position; the weakening of the screening associations after their inclusion is consistent with this possibility and should not be read as excluding an association. The models additionally adjusting for insurance/employment and self-rated health are therefore presented as sensitivity analyses rather than preferred specifications, and the fully adjusted model with the prevalence ratio and 95% confidence interval for every covariate is provided in the Supplementary Materials (Table S3). Education and income may themselves be partly consequences of long-standing limitation and are interpreted with corresponding caution.

Several survey items use skip patterns in which respondents to whom the item does not apply receive a dedicated non-applicable code. Current smoking, drinking frequency, and each treatment item are of this form, and the code carries substantive information: a respondent who has never smoked is not a current smoker, and a respondent never diagnosed is not receiving treatment. These codes were therefore assigned to the negative category. Treating them as missing would remove most respondents from adjusted models and would do so differentially by sex and disease status. The Supplementary Materials (Table S4) document each recoding decision against the survey codebook, giving the original code, its meaning in the questionnaire routing, the analytic value assigned, and the justification.

### 2.6. Statistical Analysis

All statistical analyses were conducted using IBM SPSS Statistics, Version 30 (IBM Corp., Armonk, NY, USA) and Python 3.10. All estimates accounted for the complex design using Taylor-series linearization, with the stratum and primary sampling unit identifiers supplied with the public-use files and the integrated weight divided by the number of pooled years; strata containing a single sampling unit were centered at the grand mean. Primary sampling unit identifiers are year-specific in these files and do not overlap across pooled years, whereas stratum identifiers repeat; the principal analyses were therefore repeated with strata redefined as the interaction of year and stratum. The pooled weighted population total was 44.1 million, consistent with the South Korean adult population.

Because several outcomes are common, associations are reported as prevalence ratios from survey-weighted Poisson regression with robust linearized variance rather than odds ratios, which would overstate the association for outcomes of this frequency [15,16]. Confidence intervals are referred to a t distribution with degrees of freedom equal to the number of primary sampling units minus the number of strata. The primary family for multiplicity control comprised seventeen comparisons: three disease-prevalence, four utilization and screening, one unmet-need, and nine cascade comparisons. Both unadjusted p-values and Benjamini–Hochberg q-values are reported [17]. The Benjamini–Hochberg procedure was chosen over a family-wise method such as Bonferroni because the comparisons address related but distinct outcomes within an estimation-focused analysis; false-discovery control bounds the expected proportion of false positives while retaining more power than Bonferroni for a family of this size. False-discovery control was applied to the seventeen pre-specified primary comparisons—the prevalence, utilization, preventive-screening, and unmet-need associations together with the condition-specific awareness, treatment, and control stages. The secondary analyses—effect modification, adjustment for comorbidity and for measured utilization, and the design, estimator, and specification sensitivity analyses—are descriptive or exploratory and were not included in the false-discovery family; their p-values, where reported, are unadjusted. The whole-population unmet-need estimate, the reported-need comparison, and the cumulative cascade estimates were secondary descriptive comparisons and were not included in the family.

To assess whether the measured utilization indicators altered the awareness associations, the models were refitted with outpatient visits, inpatient admissions, and general screening added, and the percentage change in the exposure coefficient on the log scale is reported. Because exposure, utilization, and outcome were measured at the same time, and because disease severity plausibly affects both utilization and detection, these changes are descriptive and are not interpreted as mediated proportions.

The design-based estimates use the standard Taylor-series linearization implemented in established survey-analysis packages. The resulting design-based estimates were verified using two complementary approaches. Linearized standard errors for five principal estimates were compared with those from a stratified delete-one-primary-sampling-unit jackknife, an independently implemented replication-based method with no shared code; the two agreed within 1.1% in every case (Table S5). The jackknife is an internal numerical check; agreement between the derived indicators and the published national statistics reported below provides an external benchmark against independently produced official estimates. Second, derived indicators were compared with published national statistics for the same survey cycle: the diabetes control rate among treated adults was 40.5% in this analysis versus a published 40.5%, and the hypercholesterolemia treatment rate among prevalent cases was 63.7% versus a published 64.8% [14]. Sensitivity analyses also replaced categorical age with a restricted cubic spline with knots at ages 25, 40, 55, 70, and 80. The analysis code is provided in Supplementary Materials File S3. As a further sensitivity analysis addressing whether the associations reflected differences in morbidity burden rather than access, the primary utilization, screening, and unmet-need models were additionally adjusted for a count of physician-diagnosed chronic conditions (hypertension, dyslipidemia, diabetes, asthma, and chronic kidney disease; range 0–5) and for obesity; because morbidity may itself be a consequence of activity limitation, this is reported as a sensitivity analysis rather than a preferred specification. The same morbidity adjustment was applied to the awareness models, with the chronic-disease count excluding the index condition, and the cancer-screening association was additionally examined within sex- and age-defined subgroups corresponding to national screening eligibility. Effect modification of the activity-limitation associations was examined for the principal outcomes (unmet need, outpatient and inpatient contact, and disease awareness) by adding multiplicative interaction terms with age group, sex, and household income to the primary models and testing them with design-based Wald tests; stratified estimates are reported where interaction was detected. Missing data were handled using complete-case analysis on an outcome-by-outcome basis, and the resulting analytic sample sizes, including those for each cascade stage, are reported in the Supplementary Materials (Table S1).

### 2.7. Ethics

The Korea National Health and Nutrition Examination Survey (KNHANES) was conducted with the approval of the Institutional Review Board of the Korea Disease Control and Prevention Agency (2022: 2018-01-03-4C-A; 2023: 2022-11-16-R-A; 2024: 2022-11-16-R-03), and written informed consent was obtained from all participants at the time of data collection.

## 3. Results

### 3.1. Sample Characteristics

Of the 16,672 respondents, 2089 reported activity limitation, corresponding to a weighted prevalence of 11.0%. Adults with activity limitation were older, less educated, and poorer: 50.9% were aged 60 years or older versus 27.6% of those without limitation, 25.4% had elementary education or less versus 9.8%, and 31.8% were in the lowest income quartile versus 12.6%. They were less often employed (46.8% versus 68.4%), more often Medical Aid beneficiaries (10.3% versus 2.6%), less often covered by private insurance (73.6% versus 88.5%), and far more likely to rate their health as poor (47.6% versus 13.9%). Their behavioral profile was not uniformly adverse: they were no more likely to be current smokers (16.0% versus 17.5%), drank considerably less (39.7% versus 57.0%), and met the aerobic activity guideline less often (38.9% versus 50.0%). Table 1 presents these comparisons.

### 3.2. Disease Burden, Utilization, Screening, and Unmet Need

Among adults aged 30 or older, activity limitation was associated with a higher prevalence of hypertension (PR 1.10, 1.04–1.17) and diabetes (1.23, 1.11–1.37); the association with hypercholesterolemia did not survive false-discovery correction (1.09, 1.01–1.17; *q* = 0.064).

Utilization and preventive uptake diverged sharply. Outpatient attendance in the preceding two weeks was reported by 45.8% of adults with activity limitation versus 22.7% of those without limitation (PR 1.75, 1.63–1.88), and inpatient admission in the preceding year by 21.0% versus 8.7% (2.20, 1.95–2.48). Participation in general health screening was 70.7% versus 73.1% (PR 0.99, 95% CI 0.95–1.02), and participation in any cancer screening among adults aged 40 or older was 68.9% versus 72.7% (PR 0.99, 95% CI 0.95–1.03); neither adjusted association was statistically detectable. The null cancer-screening association was consistent across subgroups relevant to national screening eligibility, including women aged 40 or older (0.98, 0.94–1.03), men aged 40 or older (0.99, 0.93–1.06), and adults aged 50 or older (0.97, 0.93–1.02). In the age- and sex-adjusted model, both screening estimates were lower among adults with activity limitation (PR 0.94 for each). However, both moved toward the null after further adjustment for education and household income (Table 2).

Unmet health-care need was reported by 19.0% of adults with activity limitation versus 7.7% of those without (2.39, 2.10–2.71). Almost all respondents fell into this denominator (99.9% versus 97.9%), and the whole-population estimate was accordingly almost identical (2.43, 2.14–2.76). Restricting the analysis to respondents classified as having a care need therefore had little effect on the estimated association. Table 3 presents these results. Adjusting these models for diagnosed chronic-disease burden and obesity left the associations materially unchanged: outpatient (1.69, 1.58–1.81) and inpatient (2.09, 1.86–2.35) utilization remained substantially higher, unmet need remained more than doubled (2.41, 2.12–2.73), and neither screening estimate showed a statistically detectable difference (Table S7). Adults with activity limitation carried a heavier diagnosed chronic-disease burden (mean 1.17 versus 0.67 conditions), yet additional adjustment for morbidity burden and obesity produced little change in the utilization or unmet-need associations.

### 3.3. Cascade Attainment

Awareness among prevalent cases was higher in the presence of activity limitation for all three conditions: 85.2% versus 76.0% for hypertension (1.06, 1.02–1.10), 88.5% versus 75.1% for diabetes (1.14, 1.08–1.19) and 86.2% versus 70.1% for hypercholesterolemia (1.10, 1.05–1.15), all surviving false-discovery correction. Under the biomarker-only prevalence definition, the awareness estimates were 1.16, 1.19, and 1.66, respectively, with the caveat about denominator comparability noted in the Methods. Adjusting the awareness models for comorbid chronic-disease burden (excluding the index condition) and obesity attenuated these differences toward the null (hypertension 1.02, 0.98–1.05; diabetes 1.09, 1.04–1.15; hypercholesterolemia 1.04, 1.00–1.08; Table S7). By contrast, utilization and unmet-need associations were materially unchanged under the same adjustment.

The two cascade formulations diverged at the later stages. In the secondary cumulative formulation, point estimates indicated higher treatment attainment for all three conditions (1.06, 1.11, and 1.09) and higher control for diabetes and hypercholesterolemia (1.20 and 1.10). Under the conditional formulation, no statistically detectable treatment differences emerged among those already aware (PRs 1.01, 0.99, and 1.00), and no control associations among those treated survived false-discovery correction (PRs 0.98, 1.17, and 1.01). Table 4 summarizes these estimates. As illustrated in Figure 1, the cumulative and conditional estimates were identical at the awareness stage but diverged thereafter. The cumulative treatment estimates favored adults with activity limitation across all three conditions, whereas the corresponding conditional estimates were close to the null; differences between the two formulations were also evident for the control stage, particularly for hypercholesterolemia.

The one conditional estimate approaching significance, control among treated adults with diabetes (1.17, 1.00–1.37; *q* = 0.075), was not robust to the control threshold: using glycated hemoglobin below 7.0%, the estimate was 1.07 (0.97–1.17). Among treated adults, hypercholesterolemia control was 1.01 (0.97–1.05) using total cholesterol below 200 mg/dL and 1.01 (0.95–1.09) using LDL cholesterol below 100 mg/dL.

Effect-modification analyses showed no evidence that the unmet-need association varied by age, sex, or household income. In contrast, in exploratory subgroup analyses, utilization associations were stronger among younger adults, with additional modification by sex and income; age-related modification was also observed for diabetes and hypercholesterolemia awareness (Table S8).

Adding the utilization indicators to the awareness models moved the exposure coefficient toward the null by 20.2%, 8.6%, and 7.3% for the three conditions, respectively, leaving the estimates materially unchanged (Table S9). The treatment-stage estimates showed no statistically detectable associations in any specification and are not reported separately. Given the cross-sectional design, these changes are descriptive and should not be read as evidence of mediation or as mediated proportions.

### 3.4. Sensitivity Analyses and Estimator Verification

Sensitivity analyses using alternative age modeling and survey-design specifications, together with a replication-based check of the variance estimator, left every principal estimate materially unchanged (Tables S5, S10, and S11).

## 4. Discussion

### 4.1. Principal Findings and Interpretation

South Korean adults with activity limitation had substantially higher health-care utilization but no greater preventive screening participation and markedly higher unmet health-care need. Their higher awareness of cardiometabolic disease was not accompanied by statistically detectable advantages in pharmacological treatment or biochemical control once detection was held constant. This pattern indicates that the higher awareness observed among adults with activity limitation should not be interpreted as better access to care: it attenuated toward the null after adjustment for comorbid chronic-disease burden, pointing to greater underlying morbidity and more frequent clinical contact rather than a genuine advantage in detection. The distinction matters for surveillance, because awareness counts can otherwise be mistaken for evidence of adequate care.

Cumulative and conditional cascade indicators answer different questions and may support different interpretations of where group differences arise; reporting both makes the source of apparent management differences visible.

These two formulations bear directly on how a recent finding should be read. A United States analysis using cumulative denominators reported higher awareness and higher control of hypertension among adults with disabilities [5]. The same cumulative pattern appeared here—point estimates favored the activity-limitation group at treatment and control—yet none of these later-stage differences survived once each stage was conditioned on attainment of the preceding one. The cumulative advantage therefore appears to reflect, at least in part, propagation of the detection difference rather than evidence of better management once disease is known. This is not specific to South Korea or to these conditions. Whenever two groups differ in detection, the detection difference contributes arithmetically to the later cumulative stages; when the conditional transitions between stages are similar, this can produce an apparent later-stage advantage for the better-detected group in the cumulative cascade. The two formulations therefore answer complementary questions: the cumulative cascade describes how far the diseased population reaches each stage and remains informative for population-level coverage, whereas the conditional cascade more directly evaluates whether group differences emerge in transitions between successive management stages. A cumulative-only interpretation may conflate differences in detection with differences at later management stages; reporting both therefore provides a more complete characterization of the care pathway.

Adjusting the awareness models for directly measured utilization moved the exposure coefficient only modestly toward the null—most for hypertension (−20.2%) and little for diabetes or hypercholesterolemia. Thus, adjustment for the measured utilization indicators produced only modest attenuation of the associations with awareness. Because exposure, utilization, and awareness were measured contemporaneously, this comparison is descriptive. It does not establish whether health-care contact lies on, or accounts for, a causal pathway linking activity limitation with disease awareness. Further adjustment for comorbid chronic-disease burden attenuated the awareness associations toward the null (Table S7), suggesting that differences in underlying morbidity burden may contribute to the higher awareness observed among adults with activity limitation—consistent with the alternative reading that greater awareness reflects heavier disease burden and more frequent clinical contact rather than better access. The larger biomarker-only awareness estimate for hypercholesterolemia (1.66) is not evidence of a wider true gap: excluding medication-defined cases removes many already-treated, already-aware individuals, who are more numerous in the group without limitation, so the residual biomarker-positive denominator is differentially enriched for awareness among those with limitation, and the estimate indexes denominator composition rather than access. In contrast, the association with unmet health-care need was materially unchanged after the same adjustment. Taken together, these findings indicate that the observed awareness advantage was sensitive to morbidity adjustment, whereas the disparity in unmet need was more robust to differences in measured health burden.

Two of the outcomes examined here are subjective appraisals rather than clinical events: perceived unmet need reflects the respondent’s own judgment that required care was not obtained, and awareness reflects whether a diagnosis has entered the person’s understanding of their health. That adults with activity limitation reported markedly higher unmet need while also showing higher disease awareness locates part of the disadvantage in the experience of living with and managing chronic illness rather than in access alone—the same population that is more likely to know it is ill is also more likely to report unmet need (awareness and unmet need were not analyzed jointly at the individual level). Illness perceptions and the self-regulatory appraisals that follow from them are recognized determinants of help-seeking and self-management, and tracking them alongside objective utilization may capture facets of chronic-illness experience that counts of contact do not.

### 4.2. Implications

The persistence of this disadvantage is informative in itself. South Korea combines near-universal statutory health insurance with organized national general-health and cancer-screening programs, conditions under which contact-based barriers might be expected to be small. Adults with activity limitation nonetheless reported far higher unmet need and no greater screening participation despite substantially higher outpatient and inpatient contact. Universal coverage and high utilization did not, on their own, translate into equivalent preventive uptake or lower unmet need, and monitoring health-care contact alone can therefore mask where disadvantage lies rather than locate it. Higher realized utilization and higher perceived unmet need are not opposing quantities: both can rise together with underlying need, so their co-occurrence indicates that greater realized utilization was not accompanied by lower unmet need, rather than a contradiction between the two measures. Because the design is cross-sectional, these patterns are associational. Still, they suggest that expanding health-care contact for adults with activity limitation may not, on its own, close preventive or management gaps unless screening delivery and the specific reasons for unmet need are addressed directly. Potential explanations may extend beyond insurance coverage and include transportation or physical-access difficulties, communication barriers during encounters, limited provider capacity to accommodate functional limitations, fragmented care, and the time and opportunity costs of care.

Equity monitoring should therefore track preventive-service uptake and perceived unmet need alongside health-care utilization and report both cumulative and conditional cascade indicators rather than infer adequacy of care from health-care contact alone. These mechanisms were not measured in the present study and warrant direct investigation.

### 4.3. Strengths and Limitations

Strengths include the use of a large probability sample with objectively measured biochemical and anthropometric outcomes, direct measurement of utilization, preventive screening participation and unmet need alongside cascade indicators, presentation of both cumulative and conditional cascade formulations, reporting on the prevalence-ratio scale with false-discovery control, independent verification of the linearized variance estimates using a replication-based jackknife, and concordance of derived indicators with published national statistics.

The limitations are substantial. The design is cross-sectional, so temporal ordering cannot be established, and reverse causation is a plausible alternative explanation for every association reported: cardiometabolic disease may itself produce activity limitation—the exposure item asks about restriction arising from a health problem so that morbidity may lie upstream of the limitation rather than downstream of it—and the associations with utilization and unmet need are almost certainly bidirectional. Accordingly, the associations reported here are interpreted as co-occurrence within individuals rather than as effects of activity limitation. Activity limitation was measured by a single self-reported item that conflates severity, duration, type, and cause; it does not correspond to registered disability status, and subgroup analysis by type or cause was not possible; the estimates therefore describe adults reporting activity limitation rather than any administratively defined disability population. At the population level, however, a single global activity-limitation item of this kind is a practical measure for monitoring health equity and overall care need. The KNHANES item is conceptually, but not psychometrically, comparable to the Global Activity Limitation Indicator; therefore, validity evidence established for the GALI cannot be directly applied to the single item used here [12].

Awareness, treatment, utilization, and unmet need were self-reported and subject to recall error that may differ by functional status. A count of diagnosed chronic conditions and obesity was available, and adjusting for these factors did not materially change the utilization or unmet-need associations (Table S7); finer aspects of morbidity—disease severity, treatment intensity, and adherence—could not be measured and could still contribute to the differences observed. Utilization was captured using defined recall periods. The conditional later-stage comparisons rest on progressively smaller denominators, so the absence of a treatment or control difference should be read as no statistically detectable difference rather than as demonstrated equivalence; the wider confidence intervals at these stages—diabetes control, for example, spanning 1.00 to 1.37—remain compatible with clinically meaningful differences.

Control was assessed on a single measurement occasion, and individualized lipid targets based on cardiovascular risk could not be applied. The aggregated cancer-screening item did not permit full application of cancer-specific age- and sex-eligibility criteria. The analysis is confined to the non-institutionalized population, excluding those with the most severe limitations, and respondents excluded for missing exposure were considerably older than those retained; both may have affected the reported associations. Because the non-institutionalized frame excludes those with the most severe limitations, and because the respondents excluded for missing exposure were substantially older, the underrepresentation of these groups—who may have greater health and functional needs—could plausibly attenuate rather than inflate the observed disparities, although the net direction cannot be established without knowing the joint distribution of exposure and outcome in the excluded groups. Because the utilization indicators capture only whether any visit or admission occurred, the intensity, frequency, and purpose of care could not be assessed. The survey did not record the reasons for unmet need, so it cannot be determined whether cost, transportation, physical accessibility, communication, or appointment barriers underlie the observed difference. Finally, because the South Korean program screens certain cancers below age 40, restricting the screening analysis to adults aged 40 or older imperfectly approximates eligibility; any resulting misclassification would be expected to attenuate rather than exaggerate any true difference in screening participation.

## 5. Conclusions

In this national sample, adults with activity limitation had substantially higher health-care utilization and more than double the unmet health-care need, with no advantage in screening participation and no statistically detectable advantage in cardiometabolic treatment or control once detection was held constant. Equity monitoring for this population should therefore extend beyond health-care contact and beyond cumulative management coverage to include preventive-service uptake and perceived unmet need. More broadly, because a cumulative cascade propagates differences in detection into later stages, group comparisons of chronic-disease management may benefit from reporting both cumulative and conditional attainment—the former characterizing population-level reach and the latter helping to identify whether group differences emerge at specific stage-to-stage transitions.

## Figures and Tables

**Figure 1:**
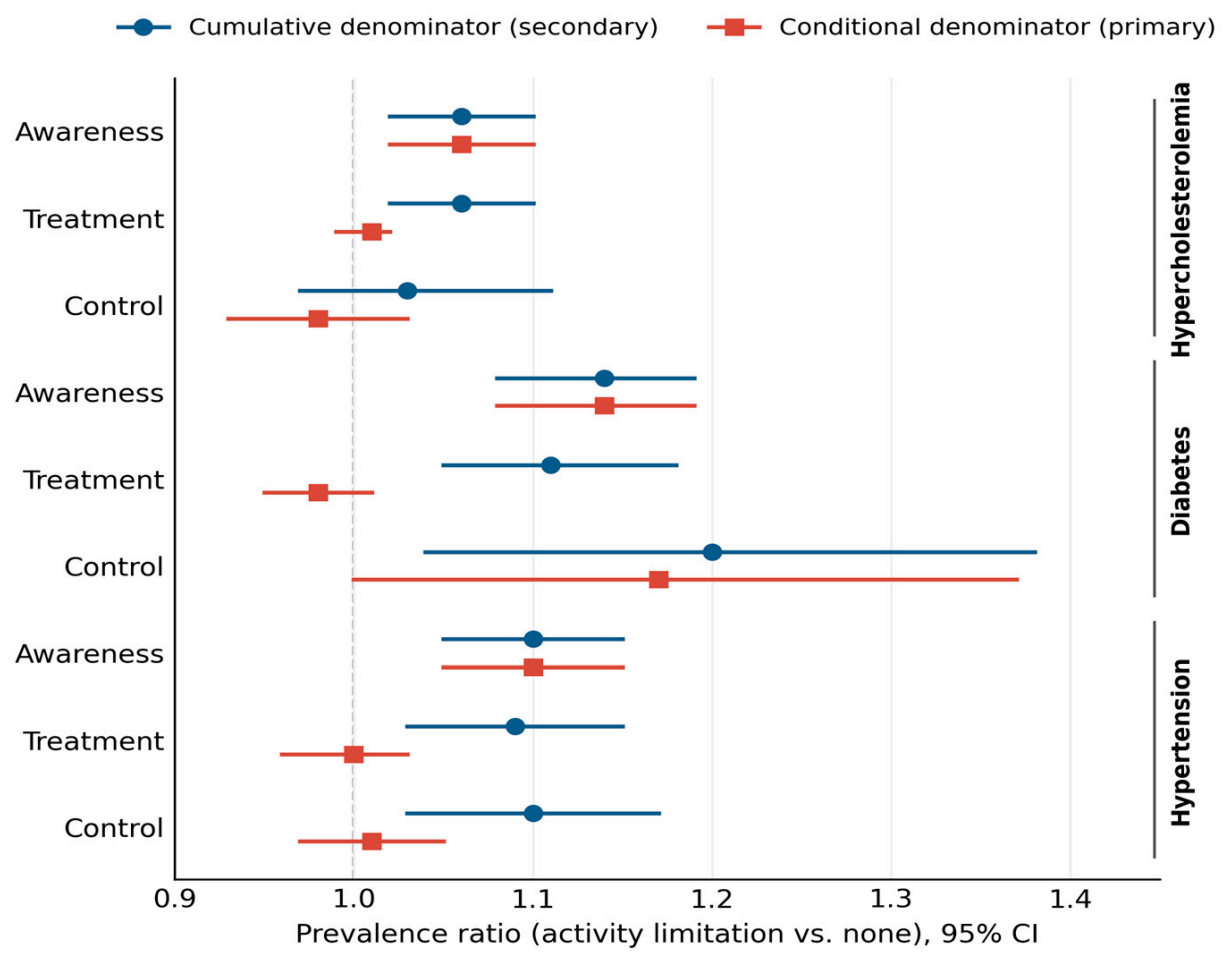
Prevalence ratios for awareness, treatment, and control among adults with activity limitation relative to those without, under cumulative (secondary) and conditional (primary) cascade denominators, for hypertension, diabetes, and hypercholesterolemia. Points are prevalence ratios and horizontal lines are 95% confidence intervals; the dashed line marks the null (PR = 1). Estimates correspond to Table 4.

**Table 1:** Weighted characteristics of South Korean adults aged 19 years or older by activity-limitation status, KNHANES 2022–2024.

Characteristic	Overall	No Limitation	Activity Limitation
Unweighted n	16,672	14,583	2089
Weighted %	100.0	89.0	11.0
Female	50.1	49.6	54.5
Age 19–39	31.9	33.5	19.0
Age 40–59	37.9	38.9	30.1
Age 60 or above	30.1	27.6	50.9
Education: elementary or less	11.5	9.8	25.4
Education: middle school	7.5	6.9	12.9
Education: high school	34.7	35.1	31.7
Education: college or above	46.2	48.3	30.1
Household income: Q1 (lowest)	15.5	12.6	31.8
Household income: Q2	22.5	22.3	24.7
Household income: Q3	29.6	30.8	22.8
Household income: Q4 (highest)	32.4	34.3	20.6
Currently employed	66.0	68.4	46.8
Medical Aid beneficiary	3.4	2.6	10.3
Private health insurance	86.9	88.5	73.6
Poor self-rated health	17.7	13.9	47.6
Current smoker	17.3	17.5	16.0
Monthly drinker	55.1	57.0	39.7
Meets aerobic activity guideline	48.8	50.0	38.9
Obesity (BMI 25 or above)	37.4	37.0	40.5

*Note*. All values are weighted percentages unless otherwise stated. BMI, body mass index; KNHANES, Korea National Health and Nutrition Examination Survey.

**Table 2:** Prevalence ratios associated with activity limitation according to stepwise covariate adjustment.

Outcome	Age, Sex	+ Education, Income	+ Smoking, Drinking ‡	+ Insurance, Employment	+ Self-Rated Health §
Unmet need among those reporting a need	2.57	2.39	2.39 (2.10–2.71)	2.60	2.23
Outpatient visit	1.79	1.77	1.75 (1.63–1.88)	1.77	1.64
Inpatient admission	2.29	2.22	2.20 (1.95–2.48)	2.18	1.93
General health screening	0.94	0.98	0.99 (0.95–1.02)	1.02	1.02
Any cancer screening, age 40+	0.94	0.98	0.99 (0.95–1.03)	1.01	1.01
Hypertension awareness	1.07	1.06	1.06 (1.02–1.10)	1.05	1.01
Diabetes awareness	1.15	1.15	1.14 (1.08–1.19)	1.14	1.09
Hypercholesterolemia awareness	1.12	1.11	1.10 (1.05–1.15)	1.10	1.06

*Note*. + indicates sequential addition of covariates to the model. ‡ Primary specification. § Sensitivity analysis only; self-rated health is plausibly a consequence of activity limitation, and adjustment may therefore result in over-control. Confidence intervals for all estimates are provided in the Supplementary Materials, Table S6.

**Table 3:** Disease burden, health-care utilization, preventive screening and unmet need by activity-limitation status.

Outcome	No Limitation, %	Activity Limitation, %	PR (95% CI)	*p*	*q*
Hypertension	32.0	47.3	1.10 (1.04–1.17)	0.001	0.003
Diabetes	14.5	24.0	1.23 (1.11–1.37)	<0.001	<0.001
Hypercholesterolemia	31.3	40.0	1.09 (1.01–1.17)	0.034	0.064
Outpatient visit, past 2 weeks	22.7	45.8	1.75 (1.63–1.88)	<0.001	<0.001
Inpatient admission, past year	8.7	21.0	2.20 (1.95–2.48)	<0.001	<0.001
General health screening	73.1	70.7	0.99 (0.95–1.02)	0.403	0.527
Any cancer screening, age 40+	72.7	68.9	0.99 (0.95–1.03)	0.565	0.629
Unmet health-care need §	7.7	19.0	2.39 (2.10–2.71)	<0.001	<0.001
whole-population definition †	7.5	19.0	2.43 (2.14–2.76)	<0.001	—
reported any care need †	97.9	99.9	1.02 (1.01–1.02)	<0.001	—

*Note.* PR, prevalence ratio from survey-weighted Poisson regression; CI, confidence interval; q, Benjamini–Hochberg false-discovery rate across the seventeen pre-specified primary comparisons. — indicates not applicable. § Primary definition, computed among respondents reporting a care need, matching the denominator of the national indicator. † Secondary comparisons, not included in the multiplicity family. Disease-prevalence rows are restricted to adults aged 30 years or older; the cancer-screening row to adults aged 40 years or older; all other rows cover adults aged 19 years or older. Models were adjusted for age group, sex, education, household income quartile, current smoking, and drinking frequency.

**Table 4:** Care-cascade attainment under cumulative and conditional denominators, adults aged 30 years or older.

Condition and Stage	*N*	No Lim, %	Alim, %	Cumulative (Secondary): PR (95% CI)	Conditional (Primary): PR (95% CI)	*q* (Conditional)
Hypertension:						
awareness	5535	76.0	85.2	1.06 (1.02–1.10)	1.06 (1.02–1.10)	0.008
treatment	4416	96.2	97.3	1.06 (1.02–1.10)	1.01 (0.99–1.02)	0.539
control	4234	79.9	75.8	1.03 (0.97–1.11)	0.98 (0.93–1.03)	0.433
Diabetes:						
awareness	2419	75.1	88.5	1.14 (1.08–1.19)	1.14 (1.08–1.19)	<0.001
treatment	1909	95.4	94.4	1.11 (1.05–1.18)	0.98 (0.95–1.01)	0.338
control	1826	39.1	45.6	1.20 (1.04–1.38)	1.17 (1.00–1.37)	0.075
Hypercholesterolemia:						
awareness	4886	70.1	86.2	1.10 (1.05–1.15)	1.10 (1.05–1.15)	<0.001
treatment	3739	89.9	91.3	1.09 (1.03–1.15)	1.00 (0.96–1.03)	0.779
control	3413	89.8	90.9	1.10 (1.03–1.17)	1.01 (0.97–1.05)	0.625

*Note*. Cumulative estimates are secondary descriptive comparisons and were not included in the multiplicity family; no multiplicity-adjusted inference was performed for them. They compute every stage against all prevalent cases, whereas the conditional estimates compute awareness among prevalent cases, treatment among those aware, and control among those treated. The awareness stage is identical under both formulations. Control is defined as blood pressure below 140/90 mmHg, glycated hemoglobin below 6.5%, and total cholesterol below 200 mg/dL. *N* is the conditional denominator (awareness among prevalent cases, treatment among those aware, and control among those treated), and the percentages are the stage-specific attainment in each group. Covariate adjustment was the same as that used in Table 2—PR, prevalence ratio; CI, confidence interval.

## Data Availability

The KNHANES public-use datasets analyzed during the current study are available from the Korea Disease Control and Prevention Agency through the KNHANES repository at https://knhanes.kdca.go.kr (accessed on 10 August 2026). File S3 provides the complete analysis code and variable-construction syntax used to reproduce the reported estimates. The original KNHANES data are not redistributed with this article.

## Supplementary Materials

The following supporting information can be downloaded at https://www.mdpi.com/article/10.3390/healthcare14193198/s1. File S1: STROBE checklist for cross-sectional studies. File S2: Supplementary tables Table S1: Sample flow for each analysis; Table S2: Response distribution and profile for the unmet-health-care-need item, pooled 2022–2024, adults aged 19 years or older; Table S3: Fully adjusted models: prevalence ratio (95% CI) for every covariate; Table S4: Coding of survey skip patterns and derived variables; Table S5: Linearized versus jackknife standard errors; Table S6: Stepwise covariate adjustment with confidence intervals; Table S7: Activity-limitation associations before and after adjustment for comorbid chronic-disease burden and obesity; Table S8: Effect modification of the activity-limitation associations; Table S9: Awareness estimates with and without additional adjustment for measured utilization; Table S10: Pooled design specification sensitivity; Table S11: Sensitivity analyses). File S3: Analysis code and derived analytic dataset.

## Abbreviations

The following abbreviations are used in this manuscript:

CI	Confidence interval
FDR	False discovery rate
GALI	Global Activity Limitation Indicator
HbA1c	Glycated hemoglobin
KNHANES	Korea National Health and Nutrition Examination Survey
LDL	Low-density lipoprotein
PR	Prevalence ratio
PSU	Primary sampling unit
STROBE	Strengthening the Reporting of Observational Studies in Epidemiology

## References

[1] Gréaux M., Moro M.F., Kamenov K., Russell A.M., Barrett D., Cieza A. (2023). Health equity for persons with disabilities: A global scoping review on barriers and interventions in healthcare services. Int. J. Equity Health.

[2] Umucu E., Vernon A.A., Pan D., Qin S., Solis G., Campa R., Lee B. (2025). Health inequities among persons with disabilities: A global scoping review. Front. Public Health.

[3] Wozniak G., Khan T., Gillespie C., Sifuentes L., Hasan O., Ritchey M., Kmetik K., Wynia M. (2015). Hypertension Control Cascade: A Framework to Improve Hypertension Awareness, Treatment, and Control. J. Clin. Hypertens..

[4] Wang J., Tan F., Wang Z., Yu Y., Yang J., Wang Y., Shao R., Yin X. (2024). Understanding Gaps in the Hypertension and Diabetes Care Cascade: Systematic Scoping Review. JMIR Public Heal. Surveill..

[5] Shah N.N., Kit B.K., Fryar C.D., Weeks J.D., Akinbami L.J. (2026). Prevalence, Awareness, and Control of Hypertension Among Adults by Disability Status, United States, August 2021–August 2023. Public Health Rep..

[6] Stevens A., Courtney-Long E., Gillespie C., Armour B.S. (2014). Hypertension Among US Adults by Disability Status and Type, National Health and Nutrition Examination Survey, 2001–2010. Prev. Chronic Dis..

[7] Park S., Stimpson J.P., Fendrick A.M. (2025). Health Care Utilization Patterns Among Adults with or Without Functional Disabilities. JAMA Netw. Open.

[8] Yang J.M., Kim J.H. (2023). The relationship between unmet medical needs awareness and health care utilization in Korean adults with social activity restriction: A secondary analysis study using propensity score matching. J. Health Res..

[9] Lee B. (2026). Determinants of Self-Reported Unmet Healthcare Needs by Disability Status: A Secondary Cross-Sectional Analysis of Linked National Survey and Administrative Data from Korea. Healthcare.

[10] Von Elm E., Altman D.G., Egger M., Pocock S.J., Gøtzsche P.C., Vandenbroucke J.P., Initiative S. (2007). The Strengthening the Reporting of Observational Studies in Epidemiology (STROBE) statement: Guidelines for reporting observational studies. Lancet.

[11] Kweon S., Kim Y., Jang M.-J., Kim Y., Kim K., Choi S., Chun C., Khang Y.-H., Oh K. (2014). Data Resource Profile: The Korea National Health and Nutrition Examination Survey (KNHANES). Int. J. Epidemiol..

[12] Van Oyen H., Bogaert P., Yokota R.T.C., Berger N. (2018). Measuring disability: A systematic review of the validity and reliability of the Global Activity Limitations Indicator (GALI). Arch. Public Heal..

[13] Madans J.H., Loeb M.E., Altman B.M. (2011). Measuring disability and monitoring the UN Convention on the Rights of Persons with Disabilities: Ehe work of the Washington Group on Disability Statistics. BMC Public Health.

[14] Korea Disease Control and Prevention Agency (2025). User Guide for the Ninth Korea National Health and Nutrition Examination Survey (KNHANES IX), 2022–2024.

[15] Zou G. (2004). A Modified Poisson Regression Approach to Prospective Studies with Binary Data. Am. J. Epidemiol..

[16] Bieler G.S., Brown G.G., Williams R.L., Brogan D.J. (2010). Estimating Model-Adjusted Risks, Risk Differences, and Risk Ratios From Complex Survey Data. Am. J. Epidemiol..

[17] Benjamini Y., Hochberg Y. (1995). Controlling the False Discovery Rate: A Practical and Powerful Approach to Multiple Testing. J. R. Stat. Soc. Ser. B Methodol..

