# Supplementary material for: Activity Limitation, Health-Care Use, and Unmet Need Among Korean Adults: Cumulative and Conditional Cardiometabolic Care Cascades
Source: Healthcare (Basel). 2026 Sep 28;14(19):3198. doi: 10.3390/healthcare14193198 (PMC13665402; doi:10.3390/healthcare14193198)
Supplement: Supplementary file 1 [file healthcare-14-03198-s001.zip › healthcare-4524007 File S1. STROBE checklist for cross-sectional studies.pdf]

File S1. STROBE Statement — checklist of items that should be included in reports of cross-sectional studies

Activity Limitation, Health-Care Use, and Unmet Need among Korean Adults: Cumulative and Conditional Cardiometabolic Care Cascades

Section	Item no.	Recommendation	Reported in
Title and abstract			
Title and abstract	1 (a)	Indicate the study's design with a commonly used term in the title or the abstract.	Title ("a national cross-sectional study" appears in the subtitle); Abstract; Methods, Design and study population (nationally representative cross-sectional survey)
	1 (b)	Provide in the abstract an informative and balanced summary of what was done and what was found.	Abstract (structured: Background, Methods, Results, Conclusions)
Introduction			
Background/rationale	2	Explain the scientific background and rationale for the investigation being reported.	Background, paragraphs 1–3
Objectives	3	State specific objectives, including any prespecified hypotheses.	Background, final paragraph
Methods			
Study design	4	Present key elements of study design early in the paper.	Methods, Design and study population, paragraph 1
Setting	5	Describe the setting, locations and relevant dates, including periods of recruitment, exposure, follow-up and data collection.	Methods, Design and study population, paragraphs 1–2
Participants	6 (a)	Give the eligibility criteria and the sources and methods of selecting participants.	Methods, Design and study population, paragraph 2
Variables	7	Clearly define all outcomes, exposures, predictors, potential confounders and effect modifiers. Give diagnostic criteria, if applicable.	Methods, Exposure; Outcomes; Covariates and coding of survey skip patterns
Data sources/measurement	8	For each variable of interest, give sources of data and details of methods of assessment. Describe comparability of assessment methods if there is more than one group.	Methods, Exposure; Cardiometabolic conditions and cascade stages; Utilization, preventive screening, and unmet need; Table S3
Bias	9	Describe any efforts to address potential sources of bias.	Methods, Covariates and coding of survey skip patterns; Outcomes (biomarker-only denominator); Statistical analysis
Study size	10	Explain how the study size was arrived at.	Methods, Design and study population, paragraph 2; Table S1
Quantitative variables	11	Explain how quantitative variables were handled in the analyses. If applicable, describe which groupings were chosen and why.	Methods, Outcomes (control thresholds); Covariates; Statistical analysis (age spline sensitivity)
Statistical methods	12 (a)	Describe all statistical methods, including those used to control for confounding.	Methods, Statistical analysis, paragraphs 1–2
	12 (b)	Describe any methods used to examine subgroups and interactions.	Methods, Statistical analysis (sex- and age-defined cancer-screening subgroup analyses); Results, Preventive care and unmet need. No formal interaction tests

Section	Item no.	Recommendation	Reported in
			were performed.
	12 (c)	Explain how missing data were addressed.	Methods, Design and study population; Covariates and coding of survey skip patterns; Tables S1–S2
	12 (d)	If applicable, describe analytical methods taking account of sampling strategy.	Methods, Statistical analysis, paragraph 1 (stratification, clustering, pooled weight, lonely-PSU handling)
	12 (e)	Describe any sensitivity analyses.	Methods, Outcomes; Statistical analysis (including a morbidity-burden sensitivity adjusting for a physician-diagnosed chronic-disease count and obesity); Table S7
Results			
Participants	13 (a)	Report numbers of individuals at each stage of study.	Methods, Design and study population; Table S1
	13 (b)	Give reasons for non-participation at each stage.	Methods, Design and study population (exposure missingness, with comparison of excluded respondents); Table S1
	13 (c)	Consider use of a flow diagram.	Table S1 provides the per-analysis sample flow in place of a flow diagram.
Descriptive data	14 (a)	Give characteristics of study participants and information on exposures and potential confounders.	Results, Sample characteristics; Table 1
	14 (b)	Indicate number of participants with missing data for each variable of interest.	Table S1
Outcome data	15	Report numbers of outcome events or summary measures.	Results, Disease burden, utilization, screening, and unmet need; Cascade attainment; Tables 3–4
Main results	16 (a)	Give unadjusted estimates and, if applicable, confounder-adjusted estimates and their precision. Make clear which confounders were adjusted for and why they were included.	Table 3 (group-specific weighted percentages and adjusted prevalence ratios); Table 4 (adjusted cumulative and conditional cascade estimates); Tables 2 and S5 (stepwise covariate-adjusted estimates with precision)
	16 (b)	Report category boundaries when continuous variables were categorized.	Methods, Outcomes (disease and control thresholds); Covariates (age, education, income categories); Table 1
	16 (c)	If relevant, consider translating estimates of relative risk into absolute risk for a meaningful time period.	Not applicable. This cross-sectional study reports prevalence ratios rather than risks over a defined follow-up period. Group-specific weighted prevalence estimates are reported in Table 3 to provide absolute measures of outcome frequency.
Other analyses	17	Report other analyses done — eg analyses of subgroups and interactions, and sensitivity analyses.	Results, Cascade attainment (cumulative formulation, biomarker-only denominator, alternative control thresholds); Sensitivity analyses and estimator verification; morbidity-burden and awareness sensitivity analyses and cancer-screening subgroups; Tables 5–6; Tables S3–S7
Discussion			
Key results	18	Summarise key results with reference to study objectives.	Discussion, paragraph 1; Conclusions

Section	Item no.	Recommendation	Reported in
Limitations	19	Discuss limitations of the study, taking into account sources of potential bias or imprecision. Discuss both direction and magnitude of any potential bias.	Discussion, Strengths and limitations
Interpretation	20	Give a cautious overall interpretation of results considering objectives, limitations, multiplicity of analyses, results from similar studies, and other relevant evidence.	Discussion, paragraphs 2–5
Generalisability	21	Discuss the generalisability (external validity) of the study results.	Discussion, Strengths and limitations (non-institutionalized population; exclusion of respondents with missing exposure)
Other information			
Funding	22	Give the source of funding and the role of the funders for the present study and, if applicable, for the original study on which the present article is based.	Declarations, Funding

Notes

The wording of the recommendations is abbreviated. The authoritative version of the checklist is available from the STROBE Initiative at https://www.strobe-statement.org and through the EQUATOR Network; the downloaded form should be used to confirm the exact wording before submission.

The Page column is left blank because pagination depends on the final typeset version. Complete it against the submitted manuscript file.

Item 16 (a): fully unadjusted regression estimates are not reported. Group-specific weighted percentages, which serve the same descriptive purpose, are given in Tables 2 and 3, and the stepwise sequence in Table 2 begins with an age- and sex-adjusted model. If the reviewers request crude estimates, they can be produced from the code in File S3.

Item 13 (c): a flow diagram was not drawn; the per-analysis sample flow is tabulated in Table S1.

Reference: von Elm E, Altman DG, Egger M, Pocock SJ, Gøtzsche PC, Vandenbroucke JP; STROBE Initiative. The Strengthening the Reporting of Observational Studies in Epidemiology (STROBE) statement: guidelines for reporting observational studies. Lancet. 2007;370(9596):1453–7.
