# Supplementary material for: Activity Limitation, Health-Care Use, and Unmet Need Among Korean Adults: Cumulative and Conditional Cardiometabolic Care Cascades
Source: Healthcare (Basel). 2026 Sep 28;14(19):3198. doi: 10.3390/healthcare14193198 (PMC13665402; doi:10.3390/healthcare14193198)
Supplement: Supplementary file 1 [file healthcare-14-03198-s001.zip › healthcare-4524007 File S2. Supplementary tables.pdf]

File S2. Supplementary tables

Activity limitation, health-care utilization, unmet need and cardiometabolic care-cascade attainment among Korean adults: a national cross-sectional study

Table S1. Sample flow for each analysis

Analysis	Start (exposure non-missing)	Excluded by age restriction	Outcome missing	Covariate missing	Final N
Hypertension prevalence	16672	1915	161	61	14535
Diabetes prevalence	16672	1915	848	49	13860
Hypercholesterolemia prevalence	16672	1915	786	49	13922
Outpatient visit	16672	0	2	64	16606
Inpatient admission	16672	0	1	64	16607
General health screening	16672	0	1015	57	15600
Cancer screening	16672	3881	809	50	11932
Unmet need among those reporting a need	16672	0	255	61	16356
Unmet health-care need, whole population	16672	0	2	65	16605

The analytic sample is 16,672 adults aged 19 years or older with non-missing activity-limitation status, drawn from 17,262 respondents with a valid integrated survey weight.

Table S2. Response distribution and profile for the unmet-health-care-need item, pooled 2022-2024, adults aged 19 years or older

Response	Original code	Unweighted n	Weighted %	Mean age	Outpatient visit in the past 2 weeks, %	Analytic value	Value in the agency-derived indicator
Needed care but did not receive it	1	1492	8.53	53.5	32.4	unmet = 1, need = 1	1
Needed care and received it	2	14938	86.98	53.6	27.6	unmet = 0, need = 1	0
Did not need care in the past year	3	254	1.81	41.8	1.2	need = 0; excluded from the primary denominator, coded 0 in the whole-population estimate	missing
Do not know / no response	9	573	2.67	66.3	0.0	missing	missing

The agency-derived indicator assigns codes 3 and 9 to missing, so the national unmet-need rate is computed among respondents reporting a care need.

Table S3. Fully adjusted models: prevalence ratio (95% CI) for every covariate.

Covariate	Unmet need	Outpatient visit	Inpatient admission
Activity limitation (vs none)	2.39 (2.10–2.71)	1.75 (1.63–1.88)	2.20 (1.95–2.48)
Age 40–59 (vs 19–39)	1.03 (0.89–1.18)	1.29 (1.18–1.40)	1.17 (1.01–1.35)
Age 60+ (vs 19–39)	0.73 (0.61–0.88)	1.62 (1.48–1.78)	1.09 (0.92–1.29)
Female (vs male)	1.44 (1.26–1.64)	1.07 (1.00–1.14)	1.13 (1.01–1.27)
Middle school (vs. elementary≤)	1.22 (0.99–1.49)	1.01 (0.92–1.11)	0.92 (0.78–1.10)
High school	1.02 (0.84–1.25)	0.85 (0.78–0.92)	0.80 (0.69–0.94)
College or above	1.05 (0.85–1.31)	0.92 (0.83–1.01)	0.71 (0.59–0.85)

Covariate	Unmet need	Outpatient visit	Inpatient admission
Income Q2 (vs Q1)	0.77 (0.65–0.91)	0.95 (0.87–1.04)	1.04 (0.90–1.21)
Income Q3	0.72 (0.60–0.86)	0.96 (0.87–1.06)	0.99 (0.84–1.18)
Income Q4 (highest)	0.57 (0.47–0.69)	1.03 (0.93–1.14)	0.97 (0.81–1.15)
Current smoker	1.31 (1.11–1.54)	0.90 (0.82–1.00)	1.06 (0.91–1.25)
Drinking: monthly	0.98 (0.86–1.12)	0.86 (0.80–0.93)	0.88 (0.77–0.99)
Drinking: weekly or more	1.05 (0.90–1.24)	0.85 (0.78–0.93)	0.89 (0.77–1.03)

Note. Single survey-weighted Poisson model per outcome (primary specification: age, sex, education, income, smoking, drinking). The activity-limitation associations remained statistically detectable after simultaneous adjustment for all covariates in the primary specification.

Table S4. Coding of survey skip patterns and derived variables

Analytic variable	Source item	Original code	Analytic value	Justification
Current smoking	BS3_1	1 daily / 2 occasional	1 (current smoker)	Substantive response
Current smoking	BS3_1	3 former smokers	0	Substantive response
Current smoking	BS3_1	8 not applicable	0 (never smoker)	Routing: item asked only of respondents with lifetime smoking; skipped respondents are never-smokers
Current smoking	BS3_1	9 don't know	missing	Non-substantive
Drinking frequency	BD1_11	1 none in past year / 2 less than monthly	0	Substantive
Drinking frequency	BD1_11	3-4 monthly to 2-4 times per month	1	Substantive
Drinking frequency	BD1_11	5-6 weekly or more	2	Substantive
Drinking frequency	BD1_11	8 not applicable	0 (lifetime abstainer)	Routing: item asked only of respondents with lifetime drinking
Hypertension treatment	DI1_2	1 medication 20+ days/month	1	National indicator definition
Hypertension treatment	DI1_2	2-5 less frequent or none	0	Substantive
Hypertension treatment	DI1_2	8 not applicable	0 (not diagnosed)	Routing: item asked only of diagnosed respondents; undiagnosed respondents are untreated
Diabetes treatment	DE1_31 / DE1_32	1 insulin or oral agent	1	Substantive
Diabetes treatment	DE1_31 / DE1_32	8 not applicable	0 (not diagnosed)	Routing, as above
Lipid treatment	DI2_2	1 medication 20+ days/month	1	National indicator definition
Lipid treatment	DI2_2	8 not applicable	0 (not diagnosed)	Routing, as above
Obesity	HE_obe	1 underweight / 2 normal / 3 pre-obese	0	BMI below 25
Obesity	HE_obe	4-6 obesity class I-III	1	BMI 25 or above
Unmet need	M_2_yr	1 need unmet	1	Substantive
Unmet need	M_2_yr	2 needs met	0	Substantive
Unmet need	M_2_yr	3 no care needed	Excluded from the primary denominator; 0 in the whole-population estimate	Matches the agency-derived indicator

Analytic variable	Source item	Original code	Analytic value	Justification
Activity limitation	LQ4_00	1 yes / 2 no	1 / 0	Substantive
Activity limitation	LQ4_00	9 don't know	missing	Non-substantive

Codes designated as not applicable arise from questionnaire routing and carry substantive information; they were assigned to the negative category rather than treated as missing.

Table S5. Linearized versus jackknife standard errors

Estimate	PR	SE, Taylor linearization	SE, stratified jackknife	Ratio jackknife / linearized
Unmet health-care need	2.428	0.0654	0.0658	1.006
Outpatient visit	1.752	0.0361	0.0363	1.004
Inpatient admission	2.201	0.0614	0.0618	1.005
Hypertension awareness	1.056	0.0189	0.0191	1.008
Diabetes awareness	1.138	0.0244	0.0247	1.011

The jackknife is a stratified delete-one-primary-sampling-unit replication estimator implemented independently of the linearization: PR, prevalence ratio; SE, standard error.

Table S6. Stepwise covariate adjustment with confidence intervals

Outcome	Model 1: age, sex	Model 2: + education, income	Model 3: + smoking, drinking (primary)	Model 4: + insurance, employment	Model 5: + self-rated health (sensitivity)
Unmet need among those reporting a need	2.57 (2.26-2.92)	2.39 (2.10-2.71)	2.39 (2.10-2.71)	2.60 (2.28-2.97)	2.23 (1.93-2.57)
Outpatient visit	1.79 (1.67-1.92)	1.77 (1.65-1.90)	1.75 (1.63-1.88)	1.77 (1.64-1.91)	1.64 (1.51-1.77)
Inpatient admission	2.29 (2.04-2.58)	2.22 (1.97-2.50)	2.20 (1.95-2.48)	2.18 (1.92-2.47)	1.93 (1.69-2.20)
General health screening	0.94 (0.91-0.97)	0.98 (0.95-1.02)	0.99 (0.95-1.02)	1.02 (0.98-1.05)	1.02 (0.99-1.06)
Any cancer screening, age 40+	0.94 (0.91-0.98)	0.98 (0.94-1.03)	0.99 (0.95-1.03)	1.01 (0.97-1.05)	1.01 (0.97-1.06)
Hypertension awareness	1.07 (1.03-1.10)	1.06 (1.02-1.10)	1.06 (1.02-1.10)	1.05 (1.01-1.09)	1.01 (0.97-1.05)
Diabetes awareness	1.15 (1.10-1.20)	1.15 (1.10-1.21)	1.14 (1.08-1.19)	1.14 (1.08-1.19)	1.09 (1.03-1.14)
Hypercholesterolemia awareness	1.12 (1.07-1.17)	1.11 (1.06-1.15)	1.10 (1.05-1.15)	1.10 (1.05-1.15)	1.06 (1.01-1.11)

Values are prevalence ratios with 95% confidence intervals for activity limitation. Model 3 is the primary specification. Model 5 is a sensitivity analysis; self-rated health is plausibly a consequence of activity limitation, and adjusting for it risks over-control.

Table S7. Activity-limitation associations before and after adjustment for comorbid chronic-disease burden and obesity.

Outcome	Primary model, PR (95% CI)	+ comorbidity & obesity, PR (95% CI)
Outpatient visit	1.75 (1.63–1.88)	1.69 (1.58–1.81)
Inpatient admission	2.20 (1.95–2.48)	2.09 (1.86–2.35)
Unmet need (need-conditional)	2.39 (2.10–2.71)	2.41 (2.12–2.73)
Hypertension awareness	1.06 (1.02–1.10)	1.02 (0.98–1.05)
Diabetes awareness	1.14 (1.08–1.19)	1.09 (1.04–1.15)
Hypercholesterolemia awareness	1.10 (1.05–1.15)	1.04 (1.00–1.08)

Note. The morbidity count comprises physician-diagnosed hypertension, dyslipidemia, diabetes, asthma, and chronic kidney disease (range 0–5), excluding the index condition, together with obesity. Utilization and unmet-need associations were

materially unchanged; awareness associations attenuated toward the null; and neither screening estimate showed a statistically detectable difference.

Table S8. Effect modification of the activity-limitation associations.

Panel A. Interaction tests (design-based Wald test p-values).

Outcome	Age group	Sex
Unmet need (need-conditional)	0.95	0.40
Outpatient visit	<0.001	0.031
Inpatient admission	<0.001	0.001
Hypertension awareness	0.083	0.153
Diabetes awareness	0.006	0.775
Hypercholesterolemia awareness	0.007	0.557

Panel B. Stratified prevalence ratios (95% CI), fully adjusted.

Outcome	Adults 19–64	Adults 65+
Unmet need	2.40 (2.05–2.82)	2.36 (1.91–2.90)
Outpatient visit	2.02 (1.83–2.23)	1.43 (1.31–1.56)
Inpatient admission	2.42 (2.05–2.84)	1.84 (1.56–2.17)
**Outcome**	**Male**	**Female**
Unmet need	2.57 (2.02–3.26)	2.26 (1.92–2.66)
Outpatient visit	1.92 (1.72–2.15)	1.62 (1.47–1.78)
Inpatient admission	2.59 (2.14–3.14)	1.90 (1.63–2.21)

Note. Interaction was tested by adding multiplicative activity-limitation × modifier terms to the primary survey-weighted Poisson models and applying a design-based Wald test. The unmet-need association showed no detectable effect modification (no evidence it differed across subgroups); utilization associations were larger in younger adults. Awareness models were fitted among prevalent cases. Interaction tests used the age categorization of the primary models (matching Table S9); the stratified estimates in Panel B additionally use a binary 19–64 versus ≥65 split, the conventional threshold distinguishing working-age from older adults, for interpretability.

Table S9. Awareness estimates with and without additional adjustment for measured utilization.

Stage	Without utilization, PR (95% CI)	With utilization, PR (95% CI)	Change in log-PR, %
Hypertension: awareness among prevalent cases	1.06 (1.02–1.10)	1.04 (1.00–1.09)	−20.2
Diabetes: awareness among the prevalent	1.14 (1.08–1.19)	1.13 (1.07–1.18)	−8.6
Hypercholesterolemia: awareness among prevalent cases	1.10 (1.05–1.15)	1.09 (1.04–1.14)	−7.3

Note. Utilization adjustment adds outpatient visits, inpatient admissions, and general health screening. A negative change indicates the coefficient moved toward the null. Because exposure, utilization, and awareness were measured contemporaneously, these changes are descriptive and not evidence of mediation. (Formerly main-text Table 5.)

Table S10. Pooled design specification sensitivity

Estimate	Strata as supplied	Strata = year x stratum
Unmet health-care need	2.388 (2.101-2.714)	2.388 (2.101-2.714)
Outpatient visit	1.752 (1.632-1.881)	1.752 (1.634-1.879)
Inpatient admission	2.201 (1.951-2.484)	2.201 (1.949-2.487)

Estimate	Strata as supplied	Strata = year x stratum
Hypertension awareness	1.056 (1.017-1.096)	1.056 (1.017-1.096)
Diabetes awareness	1.138 (1.085-1.194)	1.138 (1.086-1.194)

Primary sampling unit identifiers are year-specific in these files; stratum identifiers repeat across years. Estimates are shown with strata as supplied and with strata redefined as the interaction of survey year and stratum.

Table S11. Sensitivity analyses.

Outcome	Specification	PR (95% CI)	N
Hypertension awareness	Standard prevalence definition	1.06 (1.02–1.10)	5,535
	Biomarker-only prevalence ¶	1.16 (1.04–1.30)	2,147
Diabetes awareness	Standard prevalence definition	1.14 (1.08–1.19)	2,419
	Biomarker-only prevalence ¶	1.19 (1.10–1.27)	1,900
Hypercholesterolemia awareness	Standard prevalence definition	1.10 (1.05–1.15)	4,886
	Biomarker-only prevalence ¶	1.66 (1.18–2.32)	1,463
Diabetes control among treated	HbA1c <6.5% (primary)	1.17 (1.00–1.37)	1,826
	HbA1c <7.0%	1.07 (0.97–1.17)	1,826
Hypercholesterolemia control	Total cholesterol <200 mg/dL (primary)	1.01 (0.97–1.05)	3,413
	LDL cholesterol <100 mg/dL ‖	1.01 (0.95–1.09)	3,413
Unmet need (need-conditional)	Age categorical	2.39 (2.10–2.71)	16,356
	Age restricted cubic spline	2.40 (2.11–2.72)	16,356
	Whole-population definition (secondary)	2.43 (2.14–2.76)	16,605
Outpatient visit	Age categorical	1.75 (1.63–1.88)	16,606
	Age restricted cubic spline	1.75 (1.63–1.87)	16,606
Hypertension prevalence	Age categorical	1.10 (1.04–1.17)	14,535
	Age restricted cubic spline	1.09 (1.03–1.16)	14,535
Diabetes prevalence	Age categorical	1.23 (1.11–1.37)	13,860
	Age restricted cubic spline	1.22 (1.10–1.36)	13,860

Note. ¶ Biomarker-only prevalence excludes cases identified solely through medication use and selects for currently biomarker-positive cases; it is not directly comparable with the standard denominator. ‖ Directly measured LDL. (Formerly main-text Table 6.)
